# Enhanced Direct Photolysis of Organic Micropollutants by Far-UVC Light at 222 nm from KrCl* Excilamps

**DOI:** 10.1021/acs.estlett.3c00313

**Published:** 2023-05-26

**Authors:** Jiale Xu, Ching-Hua Huang

**Affiliations:** † Department of Civil, Construction and Environmental Engineering, North Dakota State University, Fargo, North Dakota 58102, United States; ‡ School of Civil and Environmental Engineering, Georgia Institute of Technology, Atlanta, Georgia 30332, United States

**Keywords:** KrCl* excimer lamp, UV 222 nm, Organic pollutants, Direct photolysis, Molar absorption coefficient, Quantum yield

## Abstract

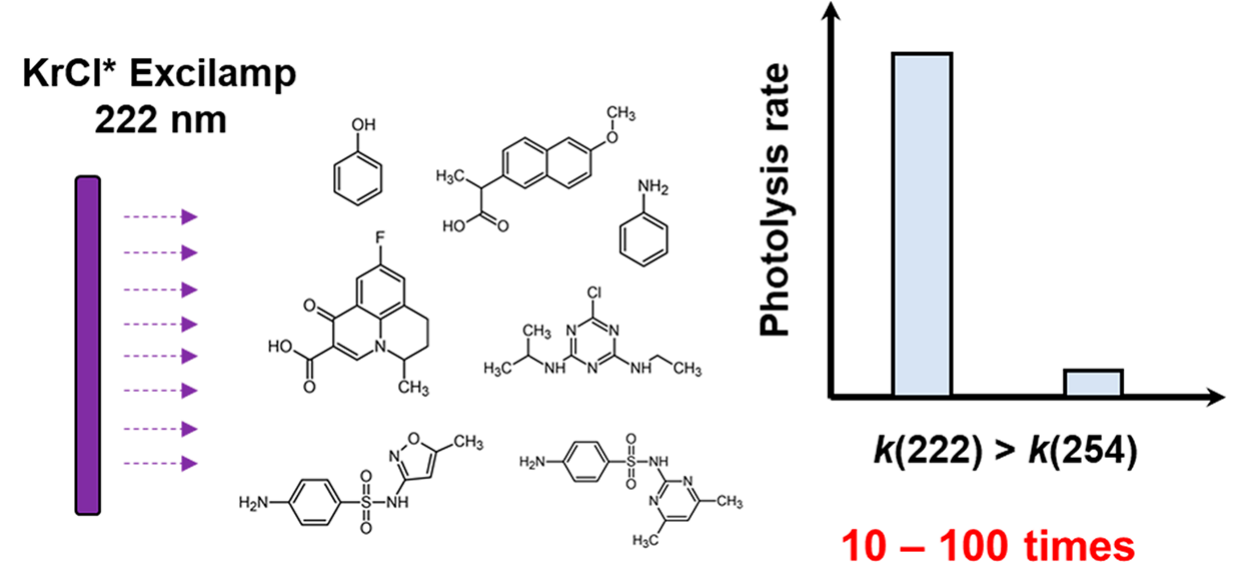

Krypton chloride (KrCl*) excilamps emitting at far-UVC 222 nm represent a promising technology for microbial disinfection and advanced oxidation of organic micropollutants (OMPs) in water treatment. However, direct photolysis rates and photochemical properties at 222 nm are largely unknown for common OMPs. In this study, we evaluated photolysis for 46 OMPs by a KrCl* excilamp and compared it with a low-pressure mercury UV lamp. Generally, OMP photolysis was greatly enhanced at 222 nm with fluence rate-normalized rate constants of 0.2–21.6 cm^2^·μEinstein^–1^, regardless of whether they feature higher or lower absorbance at 222 nm than at 254 nm. The photolysis rate constants and quantum yields were 10–100 and 1.1–47 times higher, respectively, than those at 254 nm for most OMPs. The enhanced photolysis at 222 nm was mainly caused by strong light absorbance for non-nitrogenous, aniline-like, and triazine OMPs, while notably higher quantum yield (4–47 times of that at 254 nm) occurred for nitrogenous OMPs. At 222 nm, humic acid can inhibit OMP photolysis by light screening and potentially by quenching intermediates, while nitrate/nitrite may contribute more than others to screen light. Overall, KrCl* excilamps are promising in achieving effective OMP photolysis and merit further research.

## 1 Introduction

The krypton chloride (KrCl*) excimer lamps (or excilamps) emitting a narrow-band ultraviolet light (UV) at far-UVC 222 nm have emerged as a promising disinfection technology for water treatment.^1−13^ Compared with conventional low-pressure mercury UV lamps (LPUV, emitting narrowly at 254 nm), KrCl* excilamps have enhanced disinfection performance for pathogens, especially viruses.^4−6,8−13^ For example, UV at 222 nm showed 2–4.4 times higher inactivation rates than LPUV for both single-stranded RNA viruses (e.g., MS2) and double-stranded DNA viruses (e.g., adenoviruses).^3^ KrCl* excilamps also exhibited the best performance among the available UV setups for disinfecting enveloped viruses, such as human coronavirus 229E, murine hepatitis virus, and SARS-CoV-2.^5,6^ Further, KrCl* excilamps have several additional advantages such as the absence of mercury, minimal harm to human skins and eyes,^14,15^ and stable energy output at low temperatures (<5 °C).^16^

KrCl* excilamps are also promising to be applied in advanced oxidation processes (AOPs) to remove organic micropollutants (OMPs) in water, due to increased formation of reactive oxygen species from common oxidants at 222 nm.^17−29^ Enhanced AOP by KrCl* excilamps with hydrogen peroxide (H_2_O_2_) has been reported for monochlorophenols,^20,21,26^ 4-chlorobenzoic acid,^17^ ceftriaxone,^18^ dyes (e.g., methylene blue,^19,24^ eliamine blue,^19^ and Congo red^23^), and linear alkylbenzenesulfonate.^22^ The molar absorption coefficient (ε) of H_2_O_2_ at 222 nm (ε_222_ = 99 M^–1^·cm^–1^)^30^ is 5.4 times higher than that at 254 nm (ε_254_ = 18.5 M^–1^·cm^–1^),^31^ while the quantum yield (Φ) is similar at the two wavelengths (Φ_222_ = 1.1 ± 0.1;^30^ Φ_254_ = 1.15 ± 0.05^32^). Hence, the fluence-normalized steady-state concentration of hydroxyl radical by KrCl* excilamps was reported to be 9.4 times higher than that by LPUV.^17^ Therefore, the AOP with H_2_O_2_ at 222 nm increased the degradation of 4-chlorobenzoic acid^17^ and methylene blue^19^ by 9 and 3 folds, respectively, compared to LPUV/H_2_O_2_ at similar conditions. KrCl* excilamps also enhanced AOP with persulfate.^18,27−29^ The UV222/persulfate AOP showed a removal rate of 0.78 min^–1^ for bisphenol A, 65 times greater than that (0.0118 min^–1^) obtained by LPUV/persulfate at similar conditions.^27^ Moreover, KrCl* excilamps enable de facto AOP using background water constituents such as nitrate. At 5 mg N·L^–1^ (0.36 mM), a relevant concentration in surface waters, nitrate generated 1.4 times more hydroxyl radical than H_2_O_2_ (10 mg·L^–1^; 0.29 mM) at 222 nm.^17^

Direct photolysis is potentially a critical degradation pathway for OMPs in water treatment processes (e.g., disinfection and AOPs) with KrCl* excilamps. In conventional UV setups, direct photolysis plays a minor role for most OMPs because their photolysis is weak under irradiation by LPUV (254 nm)^33^ or medium-pressure (MP) UV (200–600 nm; majority in 240–400 nm^34^).^35^ However, KrCl* excilamps may induce strong photolysis for OMPs at 222 nm for two reasons. First, photons at 222 nm feature 14% more energy than those at 254 nm, which provide more energy for molecule excitation. Second, aromatic compounds (e.g., atenolol,^36^ bisphenol A,^27^ resorcinol,^37^ and naproxen^38^) and other heteroaromatic OMPs (e.g., triazines^39^) absorb light more strongly at 222 nm than at the wavelengths of LPUV and MPUV at the same intensity. Thus far, the direct photolysis rates and fundamental photochemical properties (e.g., ε and Φ) are unknown for most OMPs at 222 nm, limiting a thorough understanding of the applicability of KrCl* excilamps in eliminating OMPs.

The goal of this study was to evaluate the direct photolysis performance by KrCl excilamps and determine ε and Φ at 222 nm and neutral pH for common OMPs. A wide range of 46 OMPs, including simple phenolic and aniline-like compounds, dyes, fluoroquinolones, triazine pesticides, sulfonamides, and several other pharmaceuticals, was selected to cover different organic structures. The photolysis rates and fundamental photochemical properties at 222 nm were compared to those at 254 nm to assess if KrCl* excilamps enhance direct photolysis of OMPs. Further, the comparison among the broad range of OMPs in their photobehaviors at the two wavelengths was drawn upon to gain mechanistic insight for the changed photolysis at 222 nm. The effect of dissolved organic matter (DOM) on OMP photolysis at 222 nm was also evaluated and compared with that at 254 nm. Lastly, the implications of this work and future research needs were commented.

## 2 Methods and Materials

### 2.1 Materials

The chemicals (Text S1) and 46 OMPs (Table S1) used in this study are provided in the Supporting Information.

### 2.2 Photolysis Experiments

A bench-scale UV collimated beam apparatus (Figure S1) with a KrCl* excilamp (Ushio) emitting mainly at 222 nm (Figure S2) was used for photolysis experiments. OMP (2 μM) solution buffered at pH 6.8 (10 mM phosphate) in Milli-Q water was irradiated under the KrCl* excilamp at room temperature (25 °C). The effective path length was determined as 1.0 cm. As a comparison, similar experiments were conducted at 254 nm with an 8 W LPUV lamp (TUV8W, Philips) in a 100 mL cylindrical quartz reactor.^40−42^ LPUV light was supplied from one side, and the effective light path length was 3.545 cm according to an approach previously described (details in Text S2).^41,42^ All solutions were magnetically stirred continuously at 300 rpm, and each OMP was tested individually. Triplicate experiments were conducted. The averaged fluence rate was measured as 31.5 and 73.7 μEinstein·m^–2^·s^–1^ for the KrCl* excilamp and LPUV setups, respectively, using iodide-iodate actinometry (details in Text S2). The fluence rate-normalized photolysis rate constant was calculated as demonstrated in Text S3 and compared between 222 and 254 nm at the path length of 1.0 cm. Molar absorption coefficients (ε, M^–1^·cm^–1^) were determined at 222 and 254 nm by triplicate experiments at pH 6.8 buffered by 10 mM phosphate. The quantum yield of OMPs was calculated as described in Text S3. The experiments with the spike of humic acid for bisphenol A are described in Text S4. The analytical methods are shown in Text S5.

## 3 Results and Discussion

### 3.1 Light Absorbance and Direct Photolysis

**Figure 1:**
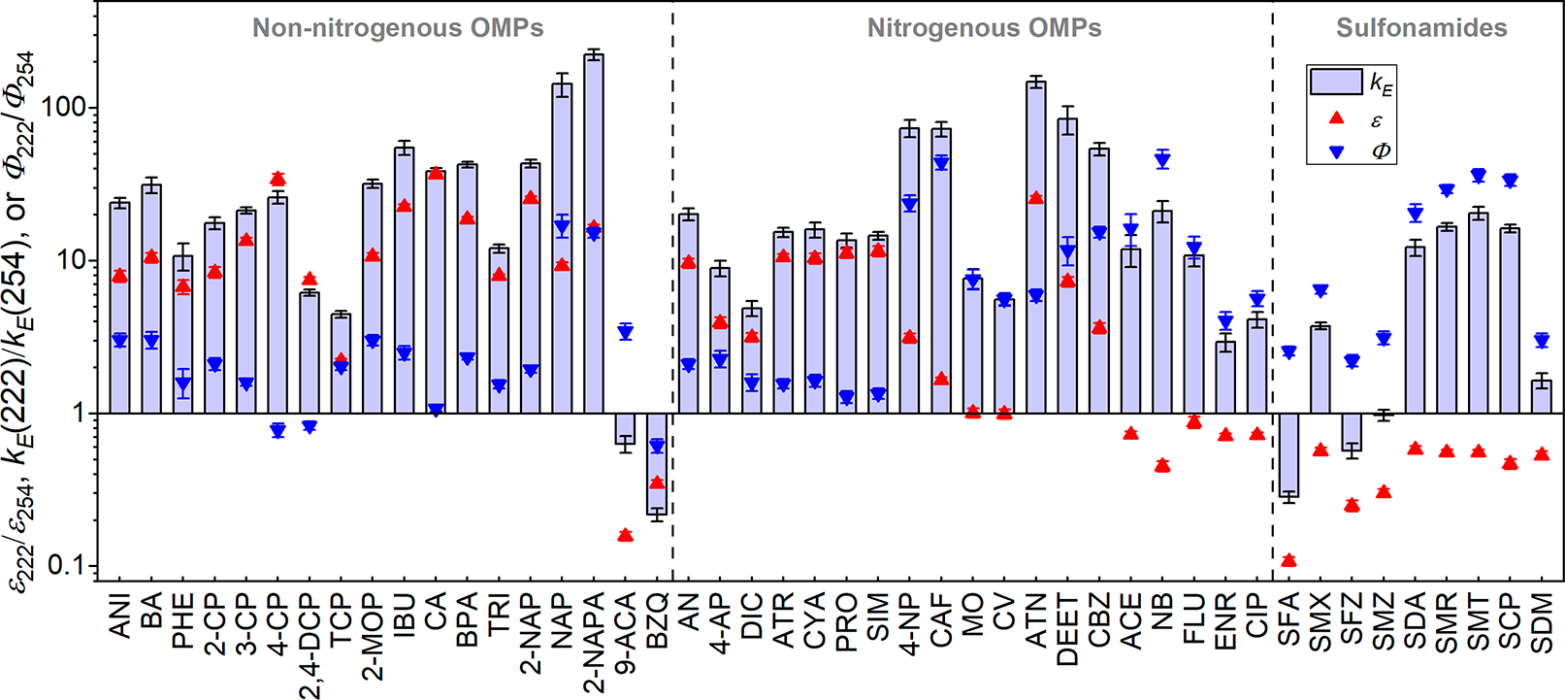
Ratios of molar absorption coefficient (ε), normalized photolysis rate constant (*k*_*E*_), and quantum yield (Φ) between 222 and 254 nm for 46 OMPs. Conditions: 2 μM OMP at pH 6.8 buffered by 10 mM phosphate, 300 rpm stirring rate, and 1.0 cm path length. *k*_*E*_(254) and Φ were calculated as described in Text S3. Structures of all 46 OMPs are shown in Table S4.

Table S3 shows the molar absorption coefficients at 222 and 254 nm for all 46 OMPs. As shown in Figure 1, most non-nitrogenous aromatic OMPs, aniline-like OMPs (i.e., AN, 4-AP, and DIC), four triazine pesticides (i.e., ATR, CYA, PRO, and SIM), 4-nitrophenol (4-NP), and four nitrogenous pharmaceuticals (i.e., CAF, ATN, DEET, and CBZ) absorbed light more strongly at 222 nm, featuring 1.7–37 times higher ε than at 254 nm. However, 9-anthracenecarboxylic acid (9-ACA), 1,4-benzoquinone (BZQ), acetaminophen (ACE), nitrobenzene (NB), three fluoroquinolones (i.e., FLU, ENR, and CIP), and nine sulfonamides exhibited lower ε at 222 nm, which was 11%–87% of that at 254 nm, indicating that the structures of these OMPs, such as fluoroquinolone and sulfonamides, feature low light absorption ability at 222 nm. Two dyes, methyl orange (MO) and crystal violet (CV), featured similar absorbance between 222 and 254 nm.

The direct photolysis of all OMPs by UV 222 nm followed pseudo-first-order kinetics, and the fluence rate-normalized decay rate constants (*k*_*E*_(222)) are summarized in Table S3. Far-UVC light at 222 nm greatly enhanced the photolysis for a wide range of OMPs, compared with LPUV (Figure 1). The *k*_*E*_(222) ranged from 0.2 to 21.6 cm^2^·μEinstein^–1^, 1.6–223 times faster than that at 254 nm, for 41 out of 46 tested OMPs. Particularly, an over 10 times higher photolysis rate at 222 nm was observed for 31 OMPs. Sulfamethizole (SMZ) showed a similar photolysis rate between 222 and 254 nm. For the other four OMPs (i.e., 9-ACA, BZQ, SFA, and SFZ); their lower absorbance at 222 nm indeed resulted in slower photolysis. However, the KrCl* excilamp still surprisingly outperformed LPUV even in treating some OMPs that absorb less light at 222 nm. For example, fluoroquinolones, six sulfonamides, ACE, and NB absorbed 13%–55% less light at 222 nm than at 254 nm, but their overall photolysis rate was still higher at 222 nm by 1.6–21 times. This observation is likely due to their higher quantum yield at 222 nm, which is further discussed in Section 3.2.

### 3.2 Quantum Yield

Direct photolysis of OMPs occurs in two major steps: (1) Organic molecules are initially activated by photons to excited states. (2) Excited states can partially undergo further reactions to form photoproducts, of which the two steps are described by two parameters, molar absorption coefficient (ε, M^–1^·cm^–1^) and quantum yield (Φ), respectively. To explore the mechanisms of enhanced OMP photolysis at 222 nm, quantum yields were determined, and the results are summarized in Figure 1 and Table S3. In this study, comparison of quantum yields and molar absorption coefficients between 222 and 254 nm was conducted to obtain preliminary mechanistic understanding for enhanced photolysis at 222 nm. Results of ε and Φ in Figure 1 suggest different mechanisms between non-nitrogenous and nitrogenous OMPs with different chemical structures.

#### 3.2.1 Non-Nitrogenous Aromatic OMPs

For most non-nitrogenous aromatic OMPs (Figure 1), the quantum yield at 222 nm was only 1.1–3.5 times more than that at 254 nm. Hence, the enhanced photolysis is mainly attributed to the stronger light absorbance at 222 nm (Figure 1); i.e., more molecules are activated to excited states. Several exceptions were observed. For naproxen (NAP) and 2-naphthoxyacetic acid (2-NAPA), two naphthyl OMPs, both molar absorption coefficient and quantum yield were 9.2–16.4 and 15–17 times higher at 222 nm than at 254 nm, respectively, suggesting that both of the two photolysis steps were enhanced at 222 nm, and naphthyl compounds are sensitive to irradiation at this wavelength. For 9-ACA, even though its quantum yield was 3.5 times higher at 222 nm than at 254 nm, the 6.4 times lower light absorbance at 222 nm still led to the slower decay (Figure 1).

BZQ, 4-chlorophenol (4-CP), and 2,4-dichlorophenol (2,4-DCP) featured lower quantum yields at 222 nm. For BZQ, both lower Φ and ε at 222 nm caused slower photolysis. For 4-CP and 2,4-DCP, the quantum yield at 222 nm was 62%–83% of that at 254 nm. A similar result was also observed previously for 4-CP.^43,44^ This phenomenon was mainly due to the varied photolysis pathways among different wavelengths. For example, chlorine detachment of 4-CP is more favorable at a wavelength closer to 266–280 nm,^43−45^ probably causing the low quantum yield at 222 nm.

#### 3.2.2 Nitrogenous OMPs and Sulfonamides

For most nitrogenous OMPs (except aniline-like OMPs and triazine pesticides), the compounds containing nitrogen functional groups, quantum yield generally played a more important role in enhancing their photolysis at 222 nm than that for non-nitrogenous OMPs. Figure 1 shows that the Φ_222_ of most nitrogenous OMPs was 4–47 times higher than Φ_254_, which contributed more than the change of absorbance to the enhanced photolysis at 222 nm. Because of the high quantum yield, nitrogenous OMPs that even absorbed less light at 222 nm, such as fluoroquinolones, still exhibited faster photolysis decay at 222 nm. To explore the reasons for the higher increase of Φ_222_ for nitrogenous OMPs, we hypothesized that this increase is related to the Φ_254_ and then compared Φ_222_/Φ_254_ and Φ_254_ and tested the correlation. Figure S3 shows a significantly negative correlation (*r* = −0.34; *p* < 0.05). Nitrogenous OMPs commonly feature lower quantum yield than non-nitrogenous OMPs at 254 nm. This low Φ_254_ for nitrogenous OMPs implied that the majority of the excited states returned to the ground state by physical reactions. However, when they were activated by high-energy photons at 222 nm, two processes may cause the high increase of Φ_222_. First, the excited states may feature a higher energy and higher possibility to undergo chemical reactions to form products. Second, different bonds may be activated by UV 222, so unique excited states were formed and more likely to undergo chemical reaction. For non-nitrogenous OMPs, the quantum yield at 254 nm was relatively high, so the effect of UV 222 was not as significant as that for nitrogenous OMPs. This hypothesis needs to be verified in future research. As for the exceptions, the quantum yield of aniline-like OMPs and triazine pesticides did not vary much between these two wavelengths, though they feature stronger absorbance at 222 nm than at 254 nm (Figure 1).

For sulfonamides (structures shown in Table S4), a group of OMPs that absorbed less at 222 nm, their heterocyclic group exerted a strong impact on the wavelength dependence of quantum yield (Figure 1). The quantum yield of sulfanilamide (SFA), a compound without the heterocyclic group, was higher than 1.0, suggesting that radical chain reactions may occur. Six-membered heterocyclic sulfonamides including SDA, SMR, SMT, and SCP showed 21–36 times increase in quantum yield at 222 nm, which was much higher than that of five-membered ring sulfonamides (i.e., SMX, SFZ, and SMZ) with only 2.2–6.5 times of increase. Hence, enhanced photolysis at 222 nm was mainly observed for six-membered heterocyclic sulfonamides. This discrepancy was likely attributed to their different photolysis mechanisms. The cleavage of the sulfonamide bond (S–N) is a dominant photolysis mechanism for five-membered ring sulfonamides,^46^ while SO_2_ extrusion is a unique pathway for six-membered ring sulfonamides.^47^ Our results suggested that the SO_2_ extrusion is potentially more sensitive to 222 nm light than the S–N bond cleavage. Moreover, the enhancement is stronger for six-membered sulfonamides with more methyl groups, i.e., Φ_222_/Φ_254_ followed SMT > SMR > SDA. Further discussion on the low Φ_222_ of sulfadimethoxine (SDM) is shown in Text S6.

It should be noted that the analysis of quantum yields and molar absorption coefficients in this section was to provide a preliminary mechanistic understanding. Using these two parameters is not enough to completely reveal the photolysis mechanism at 222 nm and the effects of structures. Future studies are required on the following aspects to provide more insights into the mechanisms of OMP photolysis. First, photolysis products from the irradiation by KrCl* excilamps should be identified and quantified using advanced analytical techniques and then compared with those from LPUV lamps. Second, the energy of different functional groups can be calculated to evaluate the photolysis efficiency for different OMPs. Third, quenchers and probes can be employed to monitor the radicals and exited states in the photolysis processes at 222 and 254 nm.

### 3.3 Effect of DOM on Direct Photolysis

UV light at 222 nm emitted by KrCl* excilamps may feature strong absorbance by DOM.^48^ Hence, we conducted a preliminary test using humic acid (HA) and bisphenol A (BPA) as model compounds to evaluate the effect of DOM at 222 nm. HA is a common DOM component in surface water,^49^ and BPA, as a typical OMP, has been widely tested for photolysis.^50−52^Figure S6 shows that the observed photolysis rate of BPA was decreased by the increase of HA concentration. At HA concentrations of 2–20 mg·L^–1^ (i.e., 0.7–7 mg C·L^–1^), the observed photolysis rate of BPA at 222 nm was decreased by 4%–58%. Light screening and quenching of excited states or radicals are the two major processes for HA to inhibit the direct photolysis of OMPs. To further assess if the decrease was caused by light screening, we calculated the photolysis rate of BPA with the consideration of background water absorption, using molar absorption coefficients of HA and BPA as well as quantum yield of BPA at 222 nm, as described in Text S4. Figure S6 shows that the calculated photolysis rate was still higher than the observed rate, suggesting that light screening is not the only factor limiting BPA photolysis. Hence, it is hypothesized that both light screening and quenching resulted in slower BPA photolysis. The roles of HA on BPA photolysis were further compared between 222 and 254 nm, as discussed in Text S7. Future research is warranted to test the quenching effect of HA from this preliminary work and assess whether it also applies to other OMPs and DOMs.

To further explore the roles of different background water constituents in light screening at 222 nm, molar absorption coefficients were first compared between 222 and 254 nm for humic acid and fulvic acid (two dominant photosensitive DOM components in natural waters^49^) and common anions. As shown in Table S5, ε was only 1.1 and 1.5 times higher at 222 nm than at 254 nm for humic acid and fulvic acid, respectively, while nitrate, nitrite, and iodide absorbed 42–916 times more, suggesting their potentially more critical roles at 222 nm. To account for real environmental scenarios, light absorbances for the five constituents were calculated using their environmentally relevant low and high concentrations. Figure S7 shows that humic acid and fulvic acid are the dominant constituents in screening UV light at 254 nm. However, nitrate and nitrite potentially emerge as the major constituents that contribute more than others in absorbing light at 222 nm. Besides light screening, the photolysis of nitrate/nitrite causes two more effects. Recent studies demonstrated that nitrate exposed to UV 222 nm was an efficient AOP in removing OMPs by generating radicals.^17,53,54^ However, nitrating agents generated from nitrate/nitrite photolysis could form toxic nitro OMPs and precursors of nitrogenous disinfection byproducts. Hence, the effects of nitrate/nitrite on OMP photolysis are complex at 222 nm. Moreover, the results based on only two specific DOMs in this study may not be applicable to other types of DOMs. Hence, future studies should focus more on the impacts of nitrogen species and different types of DOMs on OMP photolysis by KrCl* excilamps.

## 4 Environmental Implications

This study showed that KrCl* excilamps greatly enhanced the photolysis of OMPs. The direct photolysis rate constant at 222 nm ranged from 0.2 to 21.6 cm^2^·μEinstein^–1^ for 46 tested OMPs, which was 10–100 times higher than that under irradiation by a LPUV lamp at 254 nm for most OMPs. Using the KrCl* excilamp at the energy-based fluence rate of 1.7 mW·cm^–2^ (obtained from the photon-based average fluence rate of 31.5 μEinstein·m^–2^·s^–1^), the UV dose in achieving 50% concentration removal of 44 tested OMPs at 2 μM and neutral pH was only 17.3–1959 mJ·cm^–2^ (Table S3). The fluence was in the range of 17.3–110 mJ·cm^–2^ for 22 OMPs and 124–1959 mJ·cm^–2^ for 24 OMPs, which were similar to those used for wastewater and drinking water disinfection^55−57^ and for conventional AOPs,^58^ respectively. These results indicate that KrCl* excilamps can potentially achieve simultaneous OMP removal and pathogen inactivation or enhance OMP removal in AOPs at a low fluence demand. Future studies should look further into the capital cost and energy efficiency associated with the KrCl* excilamps.

Fundamental photochemical properties including molar absorption coefficient and quantum yield at 222 nm were determined for many OMPs in this study. The mechanism of enhanced photolysis at 222 nm varied among different groups of OMPs. Most non-nitrogenous OMPs, aniline-like OMPs, and triazine pesticides featured strong light absorbance at 222 nm, while their quantum yield was mostly increased by only 1.1–3.5 times, indicating that high absorbance was the driving force for the enhanced photolysis at 222 nm. However, most nitrogenous OMPs at 222 nm absorbed similarly to or less than at 254 nm. Their higher quantum yield at 222 nm was the leading factor for their faster direct photolysis. For sulfonamides, enhanced photolysis by 222 nm was mostly observed for six-membered heterocyclic compounds owing to their high quantum yield. Overall, the mechanisms preliminarily identified by this work could potentially be extended to other OMPs with similar structures. The ε and Φ values obtained in this study can be utilized in modeling photolysis under different conditions. For background water constituents, humic acid inhibited the OMPs photolysis potentially due to light screening and quenching, while nitrate/nitrite at high concentrations may play a major role in affecting OMP photolysis. Comprehensive studies are needed in future research to fully elucidate the photolysis pathways at 222 nm, evaluate the impacts of DOM and nitrate/nitrite on OMPs photolysis, and assess the contribution of direct photolysis to OMP removal compared with radical oxidation in AOPs.

## Supporting Information Available

Additional details on experimental methods and materials, structures of OMPs, data of photochemical properties for OMPs and water constituents, and supporting figures (e.g., bench-scale UV setups, light spectrum of the KrCl* excilamp, photolysis pathways for sulfachloropyridazine, photolysis rate of BPA in the presence of HA, and light absorption by water constituents) (PDF)
