# Supplementary material for: Enhanced Direct Photolysis of Organic Micropollutants by Far-UVC Light at 222 nm from KrCl* Excilamps
Source: Environ Sci Technol Lett. 2023 May 26;10(6):543–8. doi: 10.1021/acs.estlett.3c00313 (PMC10269434; doi:10.1021/acs.estlett.3c00313)
Supplement: Supplementary file 1 — ez3c00313_si_001.pdf [file ez3c00313_si_001.pdf]

Supporting Information

Enhanced Direct Photolysis of Organic Micropollutants by Far-UVC Light at 222 nm from KrCl* Excilamps

Jiale Xu[1,2,*] and Ching-Hua Huang[2,*]

[1] Department of Civil, Construction and Environmental Engineering, North Dakota State University, Fargo, North Dakota 58102, United States

[2] School of Civil and Environmental Engineering, Georgia Institute of Technology, Atlanta, Georgia 30332, United States

*Corresponding authors:

Email: jiale.xu@ndsu.edu (Jiale Xu);

Email: ching-hua.huang@ce.gatech.edu (Ching-Hua Huang)

Number of Pages: 21

Number of Texts: 7

Number of Tables: 5

Number of Figures: 7

Number of References: 19

Table of Contents

Text S1. Chemicals S3

Text S2. Determination of effective path length and average fluence rate of the KrCl* excilamp and LPUV lamp setups S3

Text S3. Calculation of quantum yield and fluence rate-normalized photolysis rate constant in buffered deionized water S4

Text S4. Determination of experimental and calculated fluence rate-normalized photolysis rate constant for bisphenol A in the presence of humic acid S7

Text S5. Analytical methods S8

Text S6. Discussion on the quantum yield of sulfadimethoxine S9

Text S7. Comparison of the roles of humic acid between 222 nm and 254 nm S9

Table S1. Organic micropollutants in this study. S11

Table S2. Wavelengths for analyzing OMPs by HPLC. S12

Table S3. Molar absorption coefficient, fluence rate-normalized photolysis rate constant, and quantum yield of 46 tested OMPs.[a] S13

Table S4. Structures of 46 OMPs investigated in this study. S14

Table S5. Photochemical properties of common water constituents. S15

Figure S1. Bench-scale UV collimated beam apparatuses with KrCl* excilamp and LPUV lamp. S16

Figure S2. Light spectrum of the KrCl* excilamp (from Ushio). S16

Figure S3. Ratio of Φ_{222}/Φ_{254} with respect to Φ_{254}. Pearson's *r* and *p*-value for correlation test are shown in the figure. S17

Figure S4. Photolysis pathways of sulfachloropyridazine (SCP). S17

Figure S5. Ratios of $\frac{\left[1\text{-}10^{-(\varepsilon_{222}C+a_{222})d}\right]}{Cd}\left(\frac{\varepsilon_{222}C}{\varepsilon_{222}C+a_{222}}\right)$ along photolysis experiments to that at initial 2 μM for BPA in the presence of 0–7 mg C/L humic acid. S18

Figure S6. Observed and calculated fluence rate-normalized photolysis rate constants for bisphenol A (BPA) with respect to humic acid (HA) concentration. Experimental conditions: 2 μM BPA at pH 6.8 buffered by 10 mM phosphate and 31.5 $\mu\text{Einstein}\cdot\text{m}^{-2}\cdot\text{s}^{-1}$ KrCl* excilamp. S18

Figure S7. Light absorption by common background water constituents at environmentally relevant concentrations at 222 nm and 254 nm. S19

Text S1. Chemicals

Acetonitrile (HPLC grade, 99.9%), boric acid (≥ 99.5%), sodium phosphate monobasic monohydrate (99.9%), and formic acid (> 98%) were purchased from Fisher Chemical. Humic acid was obtained from MP Biomedicals. Potassium iodide (99%) was obtained from Thermo Scientific Chemicals. Potassium iodate (99.5%) and sodium tetraborate decahydrate (≥ 99.5%) were purchased from Sigma-Aldrich. Sodium phosphate dibasic heptahydrate (98–102%) was purchased from EMD Millipore. Information on vendor, purity, and abbreviation of tested organic micropollutants (OMP) are listed in Table S1. All chemicals were used as received. All aqueous solutions were prepared using ultrapure Milli-Q water.

Text S2. Determination of effective path length and average fluence rate of the KrCl* excilamp and LPUV lamp setups

The effective path length of the KrCl* excilamp (Figure S1) was determined by dividing the solution volume (20 mL) by the surface area (r = 2.5 cm; $A = \pi r^2$ = 19.63 cm^2). Hence, d was 1.0 cm for the KrCl* excilamp. For the LPUV lamp setup with a cylindrical unit (Figure S1), the top circular shape was assumed to be a square shape, so the effective path length was estimated by assuming the same area between these two shapes. The effective path length was estimated to be the side length of the square as 3.545 cm, obtained from $d = \sqrt{\pi r^2}$ (r, the radius of the circle 2 cm). Our previous study[1] using model compounds and based on the volume-based fluence rate has obtained a similar path length and verified this assumption.

The average fluence rate of the KrCl* excilamp and the LPUV lamp photoreaction setups, respectively, was determined by iodide-iodate actinometry based on reaction S1[2]:

$$8I^- + IO_3^- + 3H_2O \xrightarrow{hv} 3I_3^- + 6OH^- \qquad \text{Reaction S1}$$

The Φ_λ of triiodide ion (I_3^-) was reported as around 0.94 at 222 nm and 0.72 at 254 nm in a previous study,[3] which were employed in this actinometry test for KrCl* excilamps and LPUV lamps, respectively. Briefly, an actinometer solution consisting of 0.6 M KI and 0.1 M KIO_3 in a 0.01 M $Na_2B_4O_7$ buffer was prepared. The concentration of photoproduct I_3^- exhibiting a strong absorbance in UV range was quantified by colorimetric method at λ = 352 nm with the molar absorption coefficient ε of 27636 $M^{-1}\cdot cm^{-1}$ in the 0.6 M KI/0.1 M KIO_3 solution. Based on the concentration of photoproduct I_3^-, $C(I_3^-)$ (M), the average fluence rate, $E_{avg}(\lambda)$ (Einstein·$m^{-2}\cdot s^{-1}$), was calculated by equation S1:

$$E_{avg}(\lambda) = \frac{C(I_3^-)d}{\Phi_\lambda t} \qquad \text{Equation S1}$$

where Φ_λ is the quantum yield of I_3^- at λ nm, d (m) is the effective path length, and t (s) is the reaction time. The effective path lengths for the KrCl* excilamp and the LPUV lamp setups were 1.0 cm and 3.545 cm, respectively. The same setup for OMP photolysis was used for the actinometry test, so the fluence rates already accounted for reflection factor (RF), divergence factor (DF), and petri factor (PF).

Text S3. Calculation of quantum yield and fluence rate-normalized photolysis rate constant in buffered deionized water

The kinetics of direct photolysis of OMP follows equation S2:[4]

$$-\frac{\mathrm{d}C}{\mathrm{d}t} = \frac{E_{avg}(\lambda)}{d}\Phi_\lambda\left[1\text{-}10^{-(\varepsilon_\lambda C + a_\lambda)d}\right]\left(\frac{\varepsilon_\lambda C}{\varepsilon_\lambda C + a_\lambda}\right) \qquad \text{Equation S2}$$

where C (M) is OMP concentration, t (s) is photolysis time, $E_{avg}(\lambda)$ (Einstein·$m^{-2}\cdot s^{-1}$) is the averaged fluence rate at λ nm, d (cm) is effective path length, Φ_λ (dimensionless) is the quantum yield at λ nm, ε_λ ($M^{-1}\cdot cm^{-1}$) is the molar absorption coefficient of the OMP at λ nm, and a_λ (cm^{-1})

is the absorbance by background water matrix at λ nm. In this study, absorbance by background water matrix is minimal compared with OMP (i.e., $\varepsilon_\lambda C \gg a_\lambda$), so equation S2 can be simplified to:

$$-\frac{\mathrm{d}C}{\mathrm{d}t} = \frac{E_{avg}(\lambda)}{d}\Phi_\lambda(1\text{-}10^{-\varepsilon_\lambda Cd}) \qquad \text{Equation S3}$$

The direct photolysis of OMP also followed pseudo-first-order kinetics as shown in equations S4 and S5:

$$-\frac{\mathrm{d}C}{\mathrm{d}t} = k(\lambda)C = \frac{E_{avg}(\lambda)}{d}\Phi_\lambda(1\text{-}10^{-\varepsilon_\lambda Cd}) \qquad \text{Equation S4}$$

$$k(\lambda) = E_{avg}(\lambda)\Phi_\lambda\frac{(1\text{-}10^{-\varepsilon_\lambda Cd})}{Cd} \qquad \text{Equation S5}$$

where $k(\lambda)$ (s^{-1}) is the time-based pseudo-first-order rate constant at λ nm. One constraint for this modification is that the total light absorbance (i.e., F = $1\text{-}10^{-\varepsilon_\lambda Cd}$) by OMP is less than 0.1.[5] However, for some OMPs featuring high absorbance at 222 nm or 254 nm, F is greater than 0.1, so k changes with OMP concentration along photolysis (equation S5). To obtain accurate values of k for this group of OMPs, the change of $(1\text{-}10^{-\varepsilon_\lambda Cd})/Cd$ was controlled to be less than 10% by taking samples with small changes of C. Therefore, photolysis samples were taken before 50% removal for three naphthyl OMPs (i.e., 2-NAP, NAP, and 2-NAPA) and four triazines pesticides (i.e., ATR, CYA, PRO, and SIM) at 222 nm, before 50% removal for BZQ and ACE at 254 nm, before 30% removal for nine sulfonamides (i.e., SFA, SMX, SFZ, SMZ, SDA, SMR, SMT, SCP, and SDM), three fluoroquinolones (i.e., FLU, ENR, and CIP), and two dyes (i.e., MO and CV) at 254 nm, and before 20% removal for 9-ACA at 254 nm.

Quantum yield of OMP photolysis was reported to be dependent on the initial OMP concentration.[6] In other words, quantum yield was higher at lower concentrations of OMP due to the weaker "self-quenching" process. We aimed to run experiments at OMP concentrations close

to those in real wastewater or surface water samples (commonly in nM levels[7,8]). This low OMP concentration can also maintain a small number (< 0.1) for $F = 1\text{-}10^{-\varepsilon_\lambda Cd}$ to qualify the constraint of first-order kinetics. However, due to the need to meet the instrument detection limit for analysis, 2 μM was the lowest initial concentration allowed for all OMPs for photolysis experiments. Hence, the quantum yield (Φ_λ) was calculated for OMP at 2 μM by the equation S6:

$$\Phi_\lambda = \frac{k(\lambda)}{E_{avg}(\lambda)} \frac{Cd}{(1\text{-}10^{-\varepsilon_\lambda Cd})} \qquad \text{Equation S6}$$

where C is the initial concentration 2 μM.

In the literature,[5] a simplified equation $\Phi_\lambda = \frac{k(\lambda)}{E_{avg}(\lambda)} \frac{1}{2.303\varepsilon_\lambda}$ of equation S6 was commonly used to calculate Φ_λ. However, the constraint ($F = 1\text{-}10^{-\varepsilon_\lambda Cd} < 0.1$) for this simplification was not valid for all OMPs in this study. Among the OMPs, 7 featured more than 10% absorption at 222 nm, and 17 featured more than 10% absorption at 254 nm, under the experimental conditions. Therefore, the original equation S6 was used. Although C changes in equation S6, we controlled the sampling time as mentioned above to obtain a value less than 10% for $(1\text{-}10^{-\varepsilon_\lambda Cd})/Cd$. Hence, it was valid to use the initial concentration to calculate Φ_λ, and it should be acknowledged that some of the calculated quantum yields are a close approximation.

The fluence rate-normalized photolysis rate constant $k_E(\lambda)$ ($m^2 \cdot Einstein^{-1}$) was calculated by equation S7 for experimental data to account for the different fluence rates between KrCl* excilamp and LPUV lamp:

$$k_E(\lambda) = \frac{k(\lambda)}{E_{avg}(\lambda)} \qquad \text{Equation S7}$$

Because of the different path lengths between KrCl* excilamp (1.0 cm) and LPUV lamp (3.545 cm) in the experiments, $k_E(254)$ was recalculated for LPUV lamp at effective path length of 1.0 cm using equation S8, so that it can be compared with results of the KrCl* excilamp.

$$k_E(\lambda) = \Phi_\lambda \frac{(1\text{-}10^{\text{-}\varepsilon_\lambda Cd})}{Cd} \qquad \text{Equation S8}$$

where C (M) is the initial OMP concentration (2 μM), d (m) is the effective path length (1.0 cm), ε_λ ($M^{-1}\cdot cm^{-1}$) is the molar absorption coefficient at 254 nm, and Φ_λ is the quantum yield at 254 nm. The Φ_λ at 254 was obtained through photolysis experiments using the LPUV lamp setup (d = 3.545 cm). Because of the same conditions of effective path length (1 cm) and initial OMP concentration (2 μM), it is valid to compare $k_E(\lambda)$ using the experimental results for KrCl* excilamps and the calculated results for LPUV lamps.

Text S4. Determination of experimental and calculated fluence rate-normalized photolysis rate constant for bisphenol A in the presence of humic acid

To evaluate the effect of DOM on OMP photolysis by KrCl* excilamps, experiments were conducted using bisphenol-A as the model OMP with the spike of humic acid (HA, MP Biomedicals) as the model DOM at 2–20 $mg\cdot L^{-1}$ (i.e., 0.7–7.0 $mg\ C\cdot L^{-1}$). The experimental fluence rate-normalized photolysis rate constant at 222 nm was calculated using the equation S7. To evaluate the light screening effects, the fluence rate-normalized rate was calculated with the consideration of water factor and light absorption by humic acid and bisphenol A (BPA). Assuming that the photolysis in the presence of humic acid also follows pseudo-first-order kinetics, we can obtain the following equation:

$$-\frac{\mathrm{d}C}{\mathrm{d}t} = k(222)C = \frac{E_{avg}(222)}{d}\Phi_{222}\left[1\text{-}10^{\text{-}(\varepsilon_{222}C + a_{222})d}\right]\left(\frac{\varepsilon_{222}C}{\varepsilon_{222}C + a_{222}}\right) \qquad \text{Equation S9}$$

Equations S7 and S9 can be combined into equation S10:

$$k_E(222) = \Phi_{222}\frac{\left[1\text{-}10^{\text{-}(\varepsilon_{222}C + a_{222})d}\right]}{Cd}\left(\frac{\varepsilon_{222}C}{\varepsilon_{222}C + a_{222}}\right) \qquad \text{Equation S10}$$

Based on equation S10, $k_E(222)$ is dependent on the concentration of BPA along the photolysis experiment. To test the assumption for pseudo-first-order kinetics, the combined water factor and light absorption $\frac{\left[1\text{-}10^{-(\varepsilon_{222}C + a_{222})d}\right]}{Cd}\left(\frac{\varepsilon_{222}C}{\varepsilon_{222}C + a_{222}}\right)$ was calculated for BPA concentrations from 2 μM to 0.01 μM. Figure S5 shows that the change of $\frac{\left[1\text{-}10^{-(\varepsilon_{222}C + a_{222})d}\right]}{Cd}\left(\frac{\varepsilon_{222}C}{\varepsilon_{222}C + a_{222}}\right)$ is within 3% compared with the initial condition with 2 μM. Therefore, it is valid to use initial BPA concentration of 2 μM to calculate the modeled $k_E(222)$ with the consideration of light screening in this study.

Text S5. Analytical methods

The concentrations of 44 OMP were measured by an Agilent 1100 high-performance liquid chromatography (HPLC)/diode-array detector (DAD) system equipped with an Agilent Zorbax SB-C18 column (2.1 × 150 mm, 5 μm). 4-Aminophenol was analyzed by an isocratic mobile phase of 90% of 0.1 M boric acid at pH 9.0 and 10% of acetonitrile at 0.3 $mL\cdot min^{-1}$. Other 43 OMPs were analyzed using the isocratic mobile phase: 10%–70% of acetonitrile and 90%–30% 0.1% (v/v) formic acid in water at 0.25–0.4 $mL\cdot min^{-1}$. Wavelengths for analyzing these 44 OMPs are shown in Table S2. Two dyes (CV and MO) were analyzed by colorimetric methods at 590 nm and 464 nm, respectively. The concentrations of total organic carbon (TOC) of prepared humic acid and fulvic acid solutions were measured using a Shimadzu TOC analyzer (TOC-ASI-L; Kyoto, Japan).

Text S6. Discussion on the quantum yield of sulfadimethoxine

As an exception, sulfadimethoxine (SDM) with a six-membered ring substituted by two methoxy groups showed a relatively low increase in quantum yield. Two SO_2 extrusion pathways were proposed in previous studies.[9-11] The major difference between these two pathways is the formation of an intermediate with or without a secondary amine group between the two rings (Figure S4, pathway for sulfachloropyridazine (SCP) as an example). For SMT, the pathway with intermediate 4-(2-imino-4,6-dimethylpyrimidin-1(2H)-yl)aniline (without the amine) is dominant, which was previously determined by ^{1}H-NMR spectra.[11] However, a quantum chemical study reported that the formation of an intermediate with the amine group was prevailing for SCP.[9] Similarly, this pathway was proposed for SDM.[10] To explore why the impact of 222 nm on six-membered sulfonamides was different between SDM and others, further studies are warranted using high-resolution mass spectrometry and NMR spectroscopy to elucidate the photolysis mechanism.

Text S7. Comparison of the roles of humic acid between 222 nm and 254 nm

A previous study at 254 nm reported that 1–5 mg·L^{-1} HA (Sigma Aldrich; potential carbon content 40%–56%[12-14]) improved BPA removal.[15] This discrepancy in the effects of HA at 222 nm and 254 nm is potentially due to the different roles of direct and indirect photolysis of BPA at these two wavelengths. DOM can not only screen light and quench intermediates to inhibit the direct photolysis of BPA but also sensitize the indirect photolysis of BPA.[16] At 222 nm, BPA followed a strong direct photolysis (0.667 cm^2·μEinstein^{-1}), and indirect photolysis of BPA sensitized by HA is potentially minor compared with the direct photolysis. Therefore, HA mainly inhibits the direct photolysis of BPA at 222 nm through screening and quenching, as discussed in the main

text. However, the direct photolysis at 254 nm is relatively weak (0.016 $cm^2 \cdot \mu Einstein^{-1}$; 42 times lower than that at 222 nm), so the indirect photolysis caused by HA as a sensitizer may outperform the inhibition by HA on direct photolysis, and the net effect of HA can facilitate BPA photolysis. It should be acknowledged that these hypotheses are only based on the results for BPA and HA. Further studies are indeed required to assess the roles as light screener, quencher, and sensitizer played by different DOMs at 222 nm for different OMPs.

Table S1. Organic micropollutants in this study.

#	Chemical	Abbreviation	Vendor	Purity
1	Anisole	ANI	Fisher Scientific	99%
2	Benzoic acid	BA	Sigma-Aldrich	≥ 99.5%
3	Phenol	PHE	Sigma-Aldrich	≥ 99.5%
4	2-Chlorophenol	2-CP	Alfa Aesar	99%
5	3-Chlorophenol	3-CP	Sigma-Aldrich	98%
6	4-Chlorophenol	4-CP	Sigma-Aldrich	≥ 99%
7	2,4-Dichlorophenol	2,4-DCP	Fisher Scientific	99%
8	2,4,6-Trichlorophenol	TCP	Sigma-Aldrich	98%
9	2-Methoxyphenol	2-MOP	Fisher Scientific	> 99%
10	Ibuprofen	IBU	Sigma-Aldrich	≥ 98%
11	Clofibric acid	CA	Sigma-Aldrich	≥ 98.5%
12	Bisphenol A	BPA	Sigma-Aldrich	≥ 99%
13	Triclosan	TRI	Sigma-Aldrich	–
14	2-Naphthol	2-NAP	Sigma-Aldrich	99%
15	Naproxen	NAP	Sigma-Aldrich	–
16	2-Naphthoxyacetic acid	2-NAPA	Sigma-Aldrich	98%
17	9-Anthracenecarboxylic acid	9-ACA	Fisher Scientific	> 97.0%
18	1,4-Benzoquinone	BZQ	Sigma-Aldrich	98%
19	Aniline	AN	Fisher Scientific	99.5%
20	4-Aminophenol	4-AP	Sigma-Aldrich	≥ 98%
21	Diclofenac sodium salt	DIC	Sigma-Aldrich	≥ 98%
22	Atrazine	ATR	Sigma-Aldrich	≥ 98.0%
23	Cyanazine	CYA	Supelco	Analytical standard
24	Propazine	PRO	Sigma-Aldrich	Analytical standard
25	Simazine	SIM	Sigma-Aldrich	≥ 98.0 %
26	4-Nitrophenol	4-NP	Sigma-Aldrich	≥ 99%
27	Caffeine	CAF	Sigma-Aldrich	≥ 99.0%
28	Methyl orange	MO	Fisher Scientific	> 95%
29	Crystal violet	CV	Sigma-Aldrich	≥ 90.0%
30	Atenolol	ATN	Sigma-Aldrich	≥ 98%
31	Diethyltoluamide	DEET	Sigma-Aldrich	≥ 95.0%
32	Carbamazepine	CBZ	Sigma-Aldrich	> 98%
33	Acetaminophen	ACE	Sigma-Aldrich	≥ 99.0%
34	Nitrobenzene	NB	Fisher Scientific	99.5%
35	Flumequine	FLU	Fisher Scientific	–
36	Enrofloxacin	ENR	MP Biomedicals	≥ 98%
37	Ciprofloxacin	CIP	Fisher Scientific	98%
38	Sulfanilamide	SFA	Sigma-Aldrich	≥ 98%
39	Sulfamethoxazole	SMX	Supelco	> 98.0%
40	Sulfisoxazole	SFZ	Fisher Scientific	99%
41	Sulfamethizole	SMZ	Sigma-Aldrich	≥ 99%
42	Sulfadiazine	SDA	Alfa Aesar	99%
43	Sulfamerazine	SMR	Sigma-Aldrich	≥ 99.0%
44	Sulfamethazine	SMT	Sigma-Aldrich	≥ 99.0%
45	Sulfachloropyridazine	SCP	Sigma-Aldrich	> 98%
46	Sulfadimethoxine	SDM	Sigma-Aldrich	≥ 98.5%

Table S2. Wavelengths for analyzing OMPs by HPLC.

OMP	Wavelength (nm)	OMP	Wavelength (nm)	OMP	Wavelength (nm)
ANI	225	AN	250	SFA	270
BA	210	4-AP	230	SMX	270
PHE	270	DIC	210	SFZ	270
2-CP	210	ATR	230	SMZ	270
3-CP	210	CYA	230	SDA	270
4-CP	225	PRO	230	SMR	270
2,4-DCP	210	SIM	230	SMT	270
TCP	210	4-NP	310	SCP	270
2-MOP	220	CAF	210	SDM	270
IBU	210	ATN	220		
CA	230	DEET	210		
BPA	230	CBZ	210		
TRI	210	ACE	250		
2-NAP	230	NB	270		
NAP	230	FLU	250		
2-NAPA	230	ENR	270		
9-ACA	360	CIP	270		
BZQ	250				

Table S3. Molar absorption coefficient, fluence rate-normalized photolysis rate constant, and quantum yield of 46 tested OMPs.[a]

OMP	ε_{222} ($M^{-1}\cdot cm^{-1}$)	ε_{254} ($M^{-1}\cdot cm^{-1}$)	$k_E(222)$[b] (d = 1 cm) ($cm^2\cdot\mu Einstein^{-1}$)	$k_E(254)$[b] (d = 3.545 cm) ($cm^2\cdot\mu Einstein^{-1}$)	$k_E(254)$[c] (d = 1 cm) ($cm^2\cdot\mu Einstein^{-1}$)	Φ_{222}[d] ($\times 10^{-2}$)	Φ_{254}[d] ($\times 10^{-2}$)	UV dose (50% OMP removal) ($mJ\cdot cm^{-2}$)
ANI	3755 ± 284	474 ± 20	1.559 ± 0.008	0.065 ± 0.003	0.065 ± 0.005	18.19 ± 1.37	5.97 ± 0.39	240
BA	7903 ± 498	754 ± 32	0.589 ± 0.024	0.019 ± 0.002	0.019 ± 0.002	3.30 ± 0.24	1.09 ± 0.11	635
PHE	2957 ± 277	439 ± 21	0.490 ± 0.064	0.045 ± 0.006	0.046 ± 0.007	7.24 ± 1.16	4.51 ± 0.67	764
2-CP	3678 ± 225	439 ± 25	2.240 ± 0.011	0.127 ± 0.004	0.127 ± 0.011	26.67 ± 1.62	12.59 ± 0.84	167
3-CP	4496 ± 178	334 ± 9	2.278 ± 0.009	0.106 ± 0.003	0.107 ± 0.005	22.23 ± 0.88	13.88 ± 0.52	164
4-CP	9945 ± 698	291 ± 16	14.724 ± 0.795	0.564 ± 0.001	0.565 ± 0.043	65.79 ± 5.74	84.25 ± 4.55	25
2,4-DCP	5872 ± 264	785 ± 17	2.549 ± 0.071	0.411 ± 0.009	0.413 ± 0.016	19.11 ± 1.00	22.88 ± 0.71	147
TCP	7550 ± 245	3410 ± 43	3.554 ± 0.145	0.781 ± 0.019	0.797 ± 0.024	20.81 ± 1.08	10.22 ± 0.28	105
2-MOP	5156 ± 260	482 ± 13	1.803 ± 0.017	0.056 ± 0.003	0.056 ± 0.004	15.37 ± 0.78	5.09 ± 0.30	207
IBU	8969 ± 274	399 ± 12	3.606 ± 0.050	0.065 ± 0.006	0.065 ± 0.007	17.83 ± 0.59	7.12 ± 0.71	104
CA	9651 ± 261	263 ± 9	18.443 ± 0.028	0.477 ± 0.001	0.477 ± 0.023	84.85 ± 2.24	78.80 ± 2.56	20
BPA	12669 ± 388	679 ± 20	0.667 ± 0.001	0.016 ± 0.001	0.016 ± 0.001	2.35 ± 0.07	1.00 ± 0.03	561
TRI	12030 ± 217	1511 ± 22	21.586 ± 0.812	1.776 ± 0.081	1.792 ± 0.090	80.10 ± 3.32	51.67 ± 2.48	17
2-NAP	63433 ± 1906	2500 ± 63	13.417 ± 0.316	0.305 ± 0.012	0.310 ± 0.016	10.59 ± 0.37	5.42 ± 0.25	28
NAP	46547 ± 1508	5043 ± 254	20.693 ± 2.567	0.140 ± 0.014	0.144 ± 0.018	21.45 ± 2.73	1.25 ± 0.14	18
2-NAPA	53407 ± 1503	3262 ± 129	10.145 ± 0.098	0.045 ± 0.003	0.045 ± 0.004	9.31 ± 0.25	0.61 ± 0.04	37
9-ACA	12566 ± 385	79986 ± 4607	3.011 ± 0.243	3.179 ± 0.229	4.762 ± 0.474	10.71 ± 0.92	3.09 ± 0.27	124
BZQ	4572 ± 206	13234 ± 524	3.598 ± 0.019	15.347 ± 1.269	16.555 ± 1.637	34.54 ± 1.55	56.00 ± 5.10	104
AN	6147 ± 254	636 ± 37	0.931 ± 0.030	0.046 ± 0.001	0.046 ± 0.004	6.67 ± 0.35	3.16 ± 0.18	402
4-AP	3407 ± 215	866 ± 44	2.803 ± 0.180	0.312 ± 0.021	0.314 ± 0.031	36.01 ± 3.23	15.76 ± 1.32	133
DIC	19256 ± 1109	6108 ± 198	15.247 ± 0.606	3.009 ± 0.297	3.118 ± 0.338	35.94 ± 2.44	22.48 ± 2.33	25
ATR	34392 ± 1362	3268 ± 112	5.719 ± 0.224	0.365 ± 0.014	0.372 ± 0.023	7.81 ± 0.42	4.98 ± 0.25	65
CYA	30891 ± 1001	2982 ± 215	4.708 ± 0.165	0.289 ± 0.010	0.294 ± 0.031	7.10 ± 0.33	4.32 ± 0.34	79
PRO	23924 ± 861	2136 ± 135	5.461 ± 0.237	0.395 ± 0.014	0.400 ± 0.038	10.47 ± 0.58	8.18 ± 0.59	68
SIM	33184 ± 2270	2870 ± 108	5.317 ± 0.047	0.359 ± 0.008	0.365 ± 0.021	7.50 ± 0.48	5.56 ± 0.24	70
4-NP	6517 ± 211	2084 ± 131	0.653 ± 0.057	0.009 ± 0.001	0.009 ± 0.001	4.42 ± 0.41	0.19 ± 0.01	573
CAF	6537 ± 153	3944 ± 106	0.969 ± 0.058	0.013 ± 0.001	0.013 ± 0.001	6.53 ± 0.42	0.15 ± 0.01	386
MO	7647 ± 289	7584 ± 437	0.258 ± 0.028	0.032 ± 0.002	0.034 ± 0.004	1.49 ± 0.17	0.20 ± 0.02	1452
CV	13333 ± 744	13460 ± 581	1.310 ± 0.061	0.218 ± 0.009	0.235 ± 0.017	4.40 ± 0.31	0.78 ± 0.05	286
ATN	8903 ± 256	352 ± 14	2.226 ± 0.157	0.015 ± 0.001	0.015 ± 0.001	11.08 ± 0.84	1.86 ± 0.07	168
DEET	7160 ± 374	982 ± 55	0.821 ± 0.067	0.010 ± 0.002	0.010 ± 0.002	5.06 ± 0.49	0.43 ± 0.08	456
CBZ	20454 ± 920	5651 ± 356	1.229 ± 0.035	0.022 ± 0.001	0.023 ± 0.002	2.73 ± 0.14	0.18 ± 0.01	304
ACE	5555 ± 240	7662 ± 262	0.528 ± 0.122	0.043 ± 0.001	0.044 ± 0.002	4.18 ± 0.98	0.26 ± 0.01	708
NB	2288 ± 70	5071 ± 383	0.191 ± 0.021	0.009 ± 0.001	0.009 ± 0.001	3.64 ± 0.42	0.08 ± 0.01	1959
FLU	13626 ± 1006	15589 ± 758	0.811 ± 0.111	0.068 ± 0.002	0.075 ± 0.005	2.67 ± 0.41	0.22 ± 0.01	461
ENR	12967 ± 233	18181 ± 556	5.952 ± 0.690	1.832 ± 0.108	2.031 ± 0.147	20.53 ± 2.41	5.06 ± 0.33	63
CIP	11293 ± 183	15691 ± 593	4.694 ± 0.428	1.039 ± 0.056	1.137 ± 0.085	18.52 ± 1.71	3.26 ± 0.21	80
SFA	1548 ± 45	14403 ± 907	5.661 ± 0.006	18.356 ± 0.238	19.931 ± 1.736	159.40 ± 4.62	62.11 ± 3.87	66
SMX	9063 ± 424	16015 ± 443	5.916 ± 0.029	1.444 ± 0.051	1.582 ± 0.082	28.94 ± 1.33	4.45 ± 0.20	63
SFZ	4915 ± 112	19764 ± 1601	3.389 ± 0.078	5.308 ± 0.067	5.937 ± 0.654	30.28 ± 0.97	13.65 ± 1.07	110
SMZ	5526 ± 315	18355 ± 497	1.085 ± 0.052	1.003 ± 0.060	1.113 ± 0.078	8.63 ± 0.64	2.75 ± 0.18	345
SDA	11430 ± 597	19726 ± 213	5.122 ± 0.199	0.374 ± 0.043	0.418 ± 0.049	19.98 ± 1.28	0.96 ± 0.11	73
SMR	10339 ± 369	18626 ± 335	3.763 ± 0.107	0.203 ± 0.009	0.225 ± 0.012	16.19 ± 0.73	0.55 ± 0.03	99
SMT	9507 ± 202	17140 ± 617	2.870 ± 0.178	0.127 ± 0.007	0.140 ± 0.011	13.40 ± 0.88	0.37 ± 0.02	130
SCP	11092 ± 759	23677 ± 711	4.018 ± 0.154	0.216 ± 0.006	0.247 ± 0.012	16.14 ± 1.24	0.48 ± 0.02	93
SDM	10099 ± 315	18982 ± 1093	1.082 ± 0.086	0.592 ± 0.017	0.659 ± 0.055	4.76 ± 0.40	1.57 ± 0.10	346

[a] All data was obtained for 2 μM OMP buffered at pH 6.8 by 10 mM phosphate.
[b] Obtained from experiments.
[c] Calculated using molar absorption coefficient and quantum yield for 2 μM OMP by equation S8.
[d] 2 μM OMP.

Table S4. Structures of 46 OMPs investigated in this study.

OMP	Structure	OMP	Structure	OMP	Structure
ANI	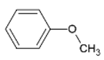	9-ACA	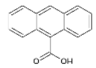	ACE	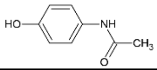
BA	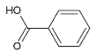	BZQ	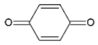	NB	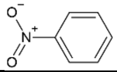
PHE	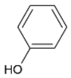	AN	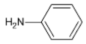	FLU	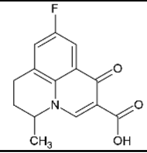
2-CP	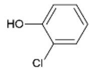	4-AP	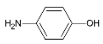	ENR	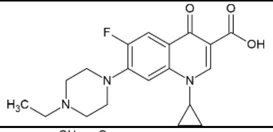
3-CP	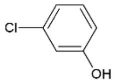	DIC	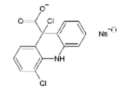	CIP	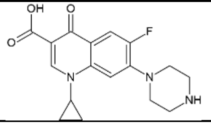
4-CP	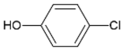	ATR	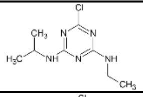	SFA	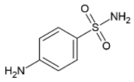
2,4-DCP	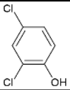	CYA	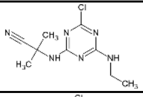	SMX	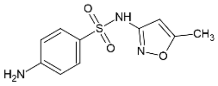
TCP	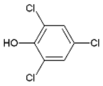	PRO	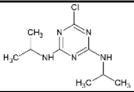	SFZ	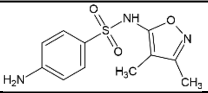
2-MOP	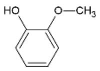	SIM	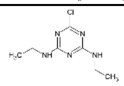	SMZ	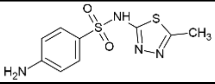
IBU	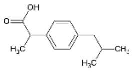	4-NP	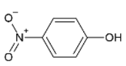	SDA	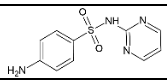
CA	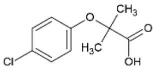	CAF	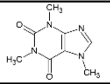	SMR	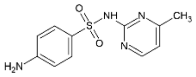
BPA	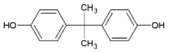	MO	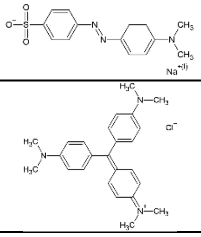	SMT	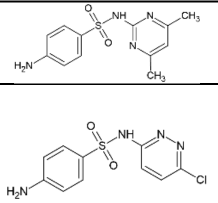
TRI	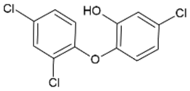	CV		SCP	
2-NAP	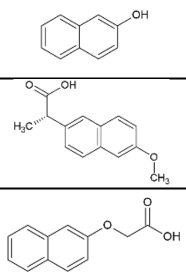	ATN	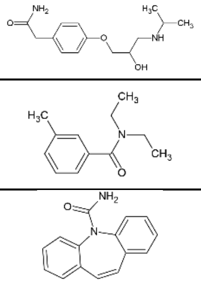	SDM	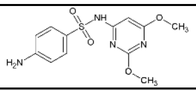
NAP		DEET		–	–
2-NAPA		CBZ		–	–

Table S5. Photochemical properties of common water constituents.

Water Constituents	ε[a]		Absorbance (cm^{-1})		
	222 nm	254 nm	Conc.[b]	222 nm	254 nm
Humic acid	29.7	26.0	1	0.030	0.026
			2	0.059	0.052
			4	0.119	0.104
Suwannee River fulvic acid	34.3	22.6	1	0.034	0.023
			2	0.069	0.045
			4	0.137	0.090
Nitrate	2747	3	2	0.392	0.0004
			10	1.962	0.0021
Nitrite	3507	12	0.2	0.050	0.0002
			1	0.251	0.0009
Iodide	11527	272	0.1	0.009	0.0002
			1	0.091	0.0021

[a] Molar absorption coefficient: $M^{-1}\cdot cm^{-1}$ is for nitrate, nitrite, and iodide; $L\cdot g\ C^{-1}\cdot cm^{-1}$ is for humic acids and fulvic acids.

[b] Environmentally relevant concentrations are 1–4 mg $C\cdot L^{-1}$ for humic acids or fulvic acids[17], 2–10 mg $N\cdot L^{-1}$ for nitrate[18], 0.2–1 mg $N\cdot L^{-1}$ nitrite[18], and 0.1–1 mg$\cdot L^{-1}$ iodide[19].

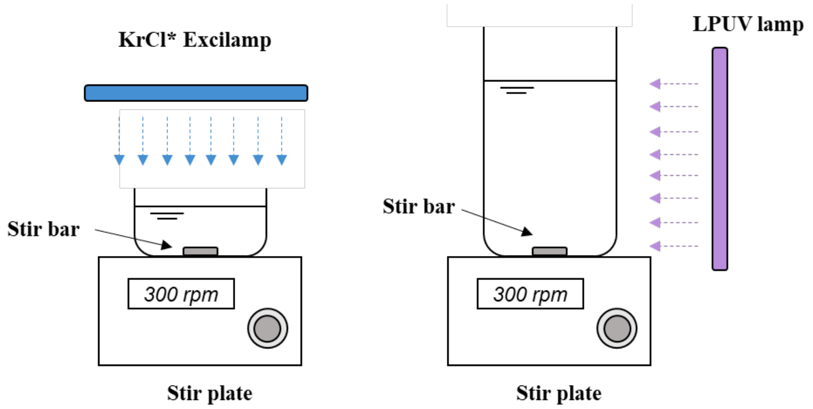

Figure S1. Bench-scale UV collimated beam apparatuses with KrCl* excilamp and LPUV lamp.

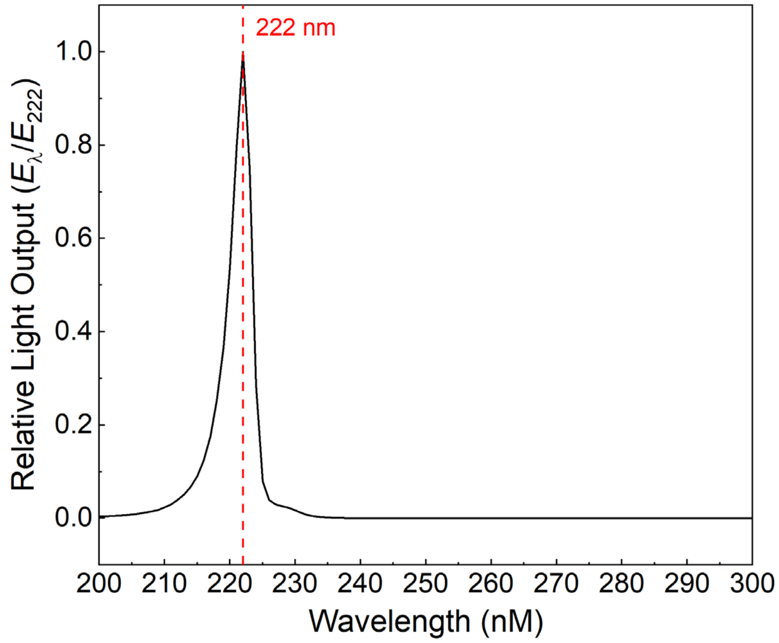

Figure S2. Light spectrum of the KrCl* excilamp (from Ushio).

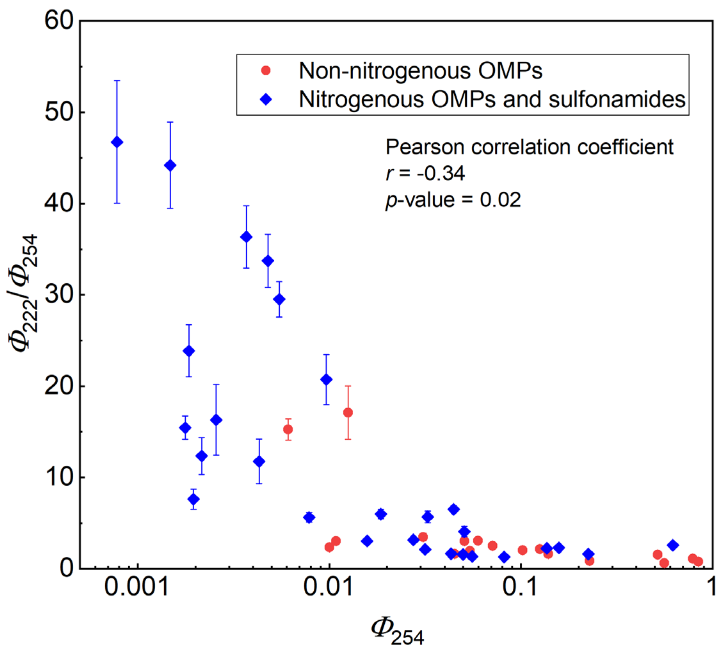

Figure S3. Ratio of Φ_{222}/Φ_{254} with respect to Φ_{254}. Pearson's r and p-value for correlation test are shown in the figure.

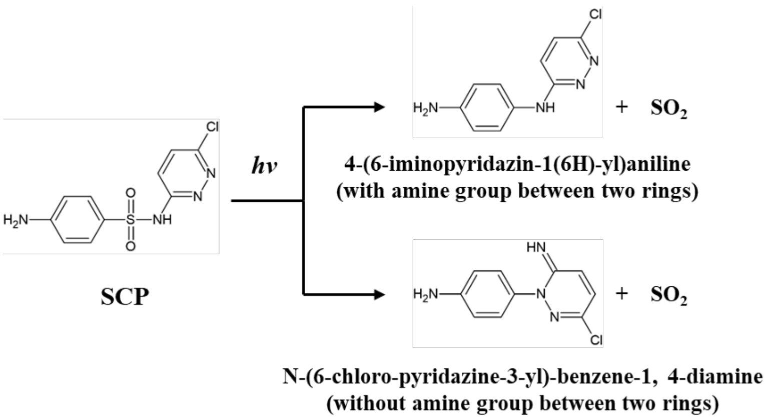

Figure S4. Photolysis pathways of sulfachloropyridazine (SCP).

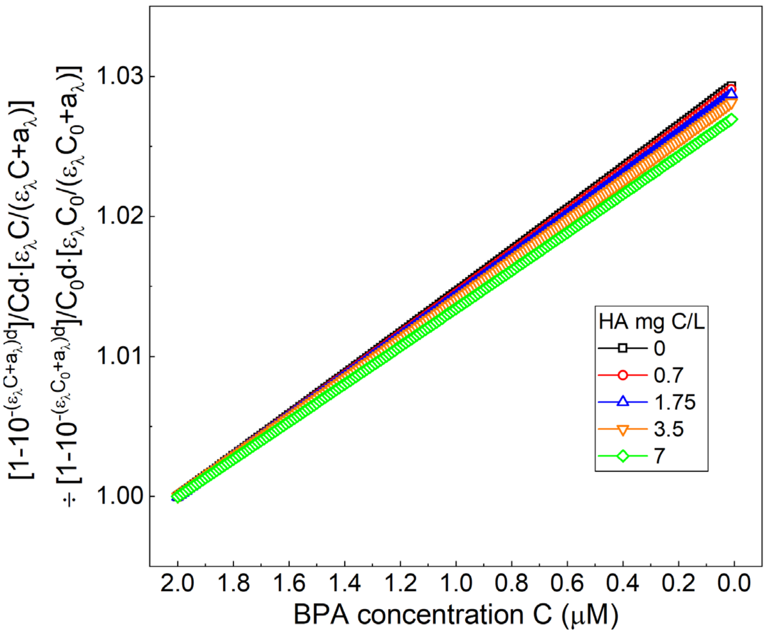

Figure S5. Ratios of $\frac{\left[1\text{-}10^{-(\varepsilon_{222}C+a_{222})d}\right]}{Cd}\left(\frac{\varepsilon_{222}C}{\varepsilon_{222}C+a_{222}}\right)$ along photolysis experiments to that at initial 2 μM for BPA in the presence of 0–7 mg C/L humic acid.

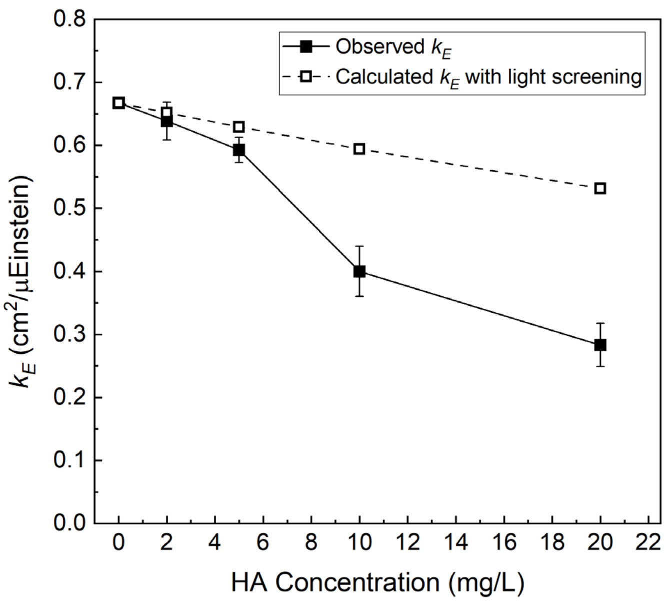

Figure S6. Observed and calculated fluence rate-normalized photolysis rate constants for bisphenol A (BPA) with respect to humic acid (HA) concentration. Experimental conditions: 2 μM BPA at pH 6.8 buffered by 10 mM phosphate and 31.5 μEinstein·m^{-2}·s^{-1} KrCl* excilamp.

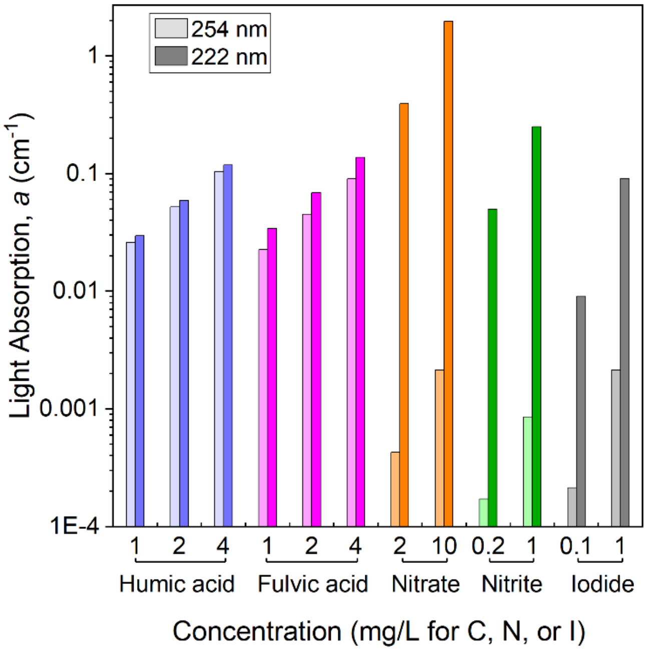

Figure S7. Light absorption by common background water constituents at environmentally relevant concentrations at 222 nm and 254 nm.

References

1. Zhang, T.; Huang, C.-H., Modeling the Kinetics of UV/Peracetic Acid Advanced Oxidation Process. *Environ. Sci. Technol.* **2020**, *54*, (12), 7579-7590. DOI: 10.1021/acs.est.9b06826.
2. Bolton, J. R.; Stefan, M. I.; Shaw, P.-S.; Lykke, K. R., Determination of the Quantum Yields of the Potassium Ferrioxalate and Potassium Iodide–Iodate Actinometers and A Method for the Calibration of Radiometer Detectors. *J. Photoch. Photobio A* **2011**, *222*, (1), 166-169. DOI: 10.1016/j.jphotochem.2011.05.017.
3. Goldstein, S.; Rabani, J., The Ferrioxalate and Iodide–Iodate Actinometers in the UV Region. *J. Photoch. Photobio A* **2008**, *193*, (1), 50-55. DOI: 10.1016/j.jphotochem.2007.06.006.
4. Bolton, J. R.; Mayor-Smith, I.; Linden, K. G., Rethinking the Concepts of Fluence (UV Dose) and Fluence Rate: The Importance of Photon-based Units - A Systemic Review. *Photochem. Photobiol.* **2015**, *91*, (6), 1252-1262. DOI: 10.1111/php.12512.
5. Zepp, R. G., Quantum Yields for Reaction of Pollutants in Dilute Aqueous Solution. *Environ. Sci. Technol.* **1978**, *12*, (3), 327-329. DOI: 10.1021/es60139a010.
6. Carlson, J. C.; Stefan, M. I.; Parnis, J. M.; Metcalfe, C. D., Direct UV Photolysis of Selected Pharmaceuticals, Personal Care Products and Endocrine Disruptors in Aqueous Solution. *Water Res.* **2015**, *84*, 350-361. DOI: 10.1016/j.watres.2015.04.013.
7. Wang, S.; Matt, M.; Murphy, B. L.; Perkins, M.; Matthews, D. A.; Moran, S. D.; Zeng, T., Organic Micropollutants in New York Lakes: A Statewide Citizen Science Occurrence Study. *Environ. Sci. Technol.* **2020**, *54*, (21), 13759-13770. DOI: 10.1021/acs.est.0c04775.
8. Ratola, N.; Cincinelli, A.; Alves, A.; Katsoyiannis, A., Occurrence of Organic Microcontaminants in the Wastewater Treatment Process. A Mini Review. *J. Hazard. Mater.* **2012**, *239-240*, 1-18. DOI: 10.1016/j.jhazmat.2012.05.040.
9. Shah, S.; Zhang, H.; Song, X.; Hao, C. Quantum Chemical Study of the Photolysis Mechanisms of Sulfachloropyridazine and the Influence of Selected Divalent Metal Ions. *Chemosphere* **2015**, *138*, 765-769. DOI: 10.1016/j.chemosphere.2015.07.068.
10. Guerard, J. J.; Chin, Y.-P.; Mash, H.; Hadad, C. M. Photochemical Fate of Sulfadimethoxine in Aquaculture Waters. *Environ. Sci. Technol.* **2009**, *43* (22), 8587-8592. DOI: 10.1021/es9020537.
11. Boreen, A. L.; Arnold, W. A.; McNeill, K. Triplet-Sensitized Photodegradation of Sulfa Drugs Containing Six-Membered Heterocyclic Groups: Identification of an SO_2 Extrusion Photoproduct. *Environ. Sci. Technol.* **2005**, *39* (10), 3630-3638. DOI: 10.1021/es048331p.
12. Rodrigues, A.; Brito, A.; Janknecht, P.; Proença, M. F.; Nogueira, R., Quantification of Humic Acids in Surface Water: Effects of Divalent Cations, pH, and Filtration. *J. Environ. Monitor.* **2009**, *11*, (2), 377-382. DOI: 10.1039/B811942B.
13. Yu, Z.-G.; Orsetti, S.; Haderlein, S. B.; Knorr, K.-H., Electron Transfer Between Sulfide and Humic Acid: Electrochemical Evaluation of the Reactivity of Sigma-Aldrich Humic Acid Toward Sulfide. *Aquat. Geochem.* **2016**, *22*, (2), 117-130. DOI: 10.1007/s10498-015-9280-0.
14. El-Ghenymy, A.; Alsheyab, M.; Khodary, A.; Sirés, I.; Abdel-Wahab, A. Corrosion Behavior of Pure Titanium Anodes in Saline Medium and Their Performance for Humic Acid Removal by Electrocoagulation. *Chemosphere* **2020**, *246*, 125674. DOI: 10.1016/j.chemosphere.2019.125674.

15. Kang, Y.-M.; Kim, M.-K.; Zoh, K.-D. Effect of Nitrate, Carbonate/Bicarbonate, Humic Acid, and H_2O_2 on the Kinetics and Degradation Mechanism of Bisphenol-A during UV Photolysis. *Chemosphere* **2018**, *204*, 148-155. DOI: 10.1016/j.chemosphere.2018.04.015.
16. Janssen, E. M. L.; Erickson, P. R.; McNeill, K. Dual Roles of Dissolved Organic Matter as Sensitizer and Quencher in the Photooxidation of Tryptophan. *Environ. Sci. Technol.* **2014**, *48* (9), 4916-4924. DOI: 10.1021/es500535a.
17. Basumallick, S.; Santra, S., Monitoring of Ppm Level Humic Acid in Surface Water Using ZnO–Chitosan Nano-Composite as Fluorescence Probe. *Appl. Water Sci.* **2017**, *7*, (2), 1025-1031. DOI: 10.1007/s13201-015-0291-1.
18. Krasner, S. W.; Westerhoff, P.; Chen, B.; Rittmann, B. E.; Nam, S.-N.; Amy, G., Impact of Wastewater Treatment Processes on Organic Carbon, Organic Nitrogen, and DBP Precursors in Effluent Organic Matter. *Environ. Sci. Technol.* **2009**, *43*, (8), 2911-2918. DOI: 10.1021/es802443t.
19. Sharma, N.; Karanfil, T.; Westerhoff, P., Historical and Future Needs for Geospatial Iodide Occurrence in Surface and Groundwaters of the United States of America. *Environ. Sci. Tech. Let.* **2019**, *6*, (7), 379-388. DOI: 10.1021/acs.estlett.9b00278.
